# Effect of a pedometer-based walking challenge on increasing physical activity levels amongst hospital workers

**DOI:** 10.1186/s13690-019-0368-7

**Published:** 2019-09-24

**Authors:** Abdulla S. Al-Mohannadi, Suzan Sayegh, Izzeldin Ibrahim, Ahmad Salman, Abdulaziz Farooq

**Affiliations:** 1 Exercise is Medicine Department, Aspetar Orthopaedic and Sports Medicine Hospital, Doha, Qatar; 2 College of Health Sciences, Qatar University, Doha, Qatar; 3 Athlete Health and Performance Research, Aspetar Orthopaedic and Sports Medicine Hospital, Doha, Qatar

**Keywords:** Health promotion, Workplace, Physical activity, Step count, Hospital staff, Quality of life

## Abstract

**Background:**

More than 50% of Qatari adults are physically inactive. The workplace is an excellent environment to implement cost-effective, efficient behavioural physical activity (PA) interventions to increase PA. This study evaluated whether a pedometer-based walking challenge would increase PA levels amongst hospital workers.

**Methods:**

A pedometer-based workplace walking intervention was implemented in April–August 2017. Amongst 800 recruited full-time hospital workers, a cross-sectional sample of 212 workers completed the online questionnaires Quality of Life Questionnaire, International Physical Activity Questionnaire (IPAQ), and Workforce Sitting Questionnaire. A sub-sample of participants (*n* = 54) wore a pedometer for 3 months. They recorded their daily step count through an online web platform linked to the pedometer. Another cross-sectional sample (*n* = 194) in the same target population completed online questionnaires at post intervention.

**Results:**

The IPAQ assessed physical activity at post-intervention was higher compared to pre-intervention. In a sub-sample (*n* = 54) that provided pedometer data, workers’ step count during intervention was significantly higher (9270) from pre-intervention (7890) (*p* = 0.048).

**Conclusions:**

Although self-reported PA was higher post-intervention, the subsample showed objectively assessed physical activity did not exceed the threshold recommended for optimal health. Therefore, encouraging participation and maintaining motivation amongst workers in a work-based PA programme is challenging.

## Background

Responsible for 9% of premature mortality worldwide [1], physical inactivity is the most prevalent modifiable risk factor for non-communicable disease. Despite being associated with an increased risk of coronary heart disease, hypertension, type 2 diabetes, obesity, and musculoskeletal disorders [1, 2], 23% of adults globally fail to meet the World Health Organisation (WHO) physical activity recommendations [3–5].

This level of physical inactivity has been shown to be dependent upon geographical region and development status of the country [3]. The WHO Eastern Mediterranean Region (EMR) has in recent times seen rapid economic development and industrialization. Paralleled with this growth has been a soar in the rate of non-communicable diseases, and notably demonstrating the highest rate of physical inactivity worldwide, with a 10% increase on the global average [5]. Specifically within the region, countries within the Gulf Corporation Council highlight the magnitude of the problem with rates ranging from 46 to 96% [6].

There are several reasons which could explain the high levels physical inactivity including cultural restrictions, social factors and the subtropical desert climes that are characterised by low annual rainfall and intensely hot and humid summers [7]. It is therefore important to promote health and to engage people in physical activity and positive lifestyle behaviours, and the workplace is one such potential environment to do so. Evidence in the literature indicates a dose-response relationship between PA and positive work performance, fewer sick days, and decreases absenteeism [8] and for the past 3 decades, PA programmes have been the cornerstone of several workplace health promotion programmes [9] having demonstrated their ability to significantly improve in health outcomes such as fitness, lipids, PA behaviour and workplace outcomes such as improvements in attendance, increased productivity and lower job stress [10]. However, the focus of research to date has not been on predictors of employee participation in PA. The aim of this study was therefore to evaluate the impact of a 3-month workplace walking challenge on PA levels, for workers in a hospital setting.

## Methods

### Study design and population

**Fig. 1:**
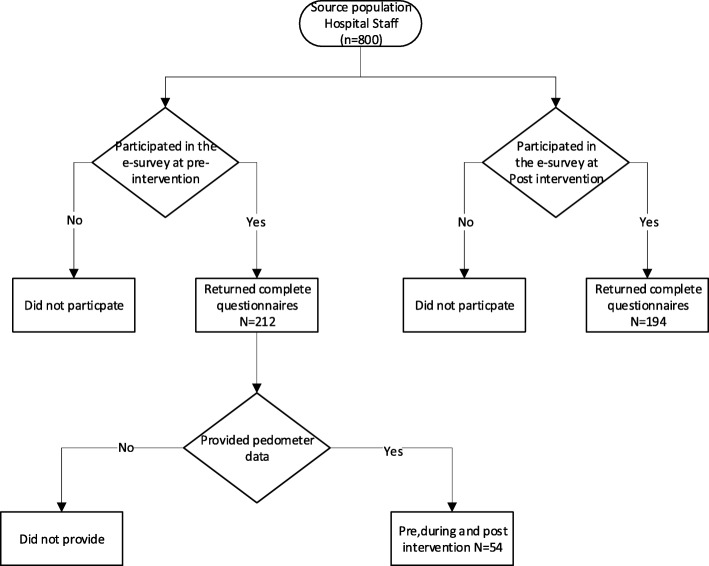
A flow diagram for study sampling

Two cross-sectional surveys were conducted at pre (October 2016) and post-intervention (February 2017). A sub sample of those that provided complete pedometer data were included for a longitudinal follow up (November 2016 to January 2017). The study was conducted at Aspetar, Orthopaedic and Sports Medicine Hospital (Doha, Qatar). Eight hundred male and female hospital staff working in clinical or nonclinical settings (aged ≥18 years) were initially invited to participate. (Fig. 1).

The target population were recruited via corporate email through the hospital’s marketing department. The email invited them to join the study by completing an online questionnaire. The email explained the objective of the study. The pre-intervention and post-intervention surveys were sent to all 800 office workers. The collection process of all data was anonymised. No personal information was collected that could identify a participant. Eventually, 212 participants returned the online questionnaire with complete data at pre-intervention and 194 at post-intervention and thus met the criteria for data analysis. (Fig. 1).

### Intervention

This study used a nation-wide ongoing community programme for PA promotion in the State of Qatar, called Step into Health [11]. Details about the programme were announced through the hospital network via billboards and social media. The programme primarily promotes behavioural change and empowers self-management of PA. All hospital staff were encouraged to join the programme and were provided with support to register. Individuals who register for the Step in Health programme received a pocket-sized pedometer (HJ-324 U; Omron Corp., Kyoto, Japan). They could opt to upload their pedometer data through an online platform (www.stepintohealth.qa). Registered members could also choose to share their data by joining online groups, and members of the hospital community group were included as a subsample and analysed separately.

The 212 included participants provided consent electronically and could withdraw at any stage. Anonymised background information was self-reported online (Table 1).

**Table 1:** Background information of the study participants at pre and post intervention^b^

Variable	Cross-sectional sample Pre (*n* = 212) n(%)	Cross-sectional sample Post (*n* = 194) n(%)	Subsample with pedometer data (*n* = 54) ^a^ n(%)
Gender			
Female	64 (30.2)	69 (35.6)	23 (43.4)
Male	148 (69.8)	125 (64.4)	30 (56.6)
Age group (years)			
35 and below	43 (20.3)	36 (18.6)	15 (28.3)
35–44	95 (44.8)	82 (42.3)	12 (22.6)
45–54	57 (26.9)	54 (27.8)	21 (39.6)
55 and above	17 (8.0)	22 (11.3)	5 (9.4)
Marital status			
Married	178 (84.0)	163 (84.0)	24 (88.9)
Single	34 (16.0)	31 (16.0)	3 (11.1)
Height (cm) Mean ± SD	171.2 ± 17.8	168.6 ± 27.4	167.7 ± 9.5
Weight (kg) Mean ± SD	79.1 ± 16.3	77.9 ± 15.9	75.2 ± 17.9
Ever Smoked			
No	115 (54.2)	107 (55.2)	20 (74.1)
Yes	97 (45.8)	87 (44.8)	7 (25.9)
If yes, frequency of smoking			
No	93 (60.0)	99 (66.0)	26 (96.3)
Rarely	21 (13.5)	16 (10.7)	0 (0)
Sometimes	27 (17.4)	25 (16.7)	1 (3.7)
Most of the time	14 (9.0)	10 (6.7)	0 (0)
Ethnicity			
Arab	77 (36.3)	70 (36.1)	20 (37.7)
Asian/Pacific Islander	31 (14.6)	27 (13.9)	19 (35.8)
Black	2 (0.9)	4 (2.1)	0 (0)
Hispanic or Latino	5 (2.4)	1 (0.5)	1 (1.9)
Other	5 (2.4)	5 (2.6)	0 (0)
White	92 (43.4)	87 (44.8)	13 (24.5)
Education level			
High school graduate	17 (8.0)	10 (5.2)	1 (3.7)
Diploma or the equivalent	30 (14.2)	39 (20.1)	5 (18.5)
Bachelor’s degree	80 (37.7)	69 (35.6)	12 (44.4)
Master’s degree	49 (23.1)	34 (17.5)	5 (18.5)
Professional degree	12 (5.7)	12 (6.2)	0 (0.0)
Doctorate degree	24 (11.3)	30 (15.5)	4 (14.8)

^a^Not all subsample with pedometer data *n* = 54 provided this information, percentages are computed based on valid counts

^b^No statistical differences in participant’s characteristics at pre vs post intervention

The 3-month workplace challenge was promoted through internal announcements. They received health tips through automated emails and mobile text messages throughout the challenge. Participants who averaged 10,000 steps per day were randomly selected to receive incentives at the end of the 3-month challenge. The weekly top walkers were announced internally to all staff. In accordance with Tudor-Locke and Bassett [12] the following public health ranges for pedometer count were used: 5000–7499 steps as ‘low active’; 7500–10,000 steps as ‘moderately active’; and > 10,000 steps as ‘active’.

Omron pedometers (model HJ-324 U) were previously validated, have an absolute percent error of < 3.0% and a coefficient of variation of < 2.1% [13, 14]. During the study period, outdoor environmental conditions—specifically temperature and wind—remained relatively stable.

### Questionnaires

The Health Survey Short Form-36, version 2 (SF-36v2) [15], the short version of International Physical Activity Questionnaire (IPAQ), and the Workforce Sitting Questionnaire (WSQ) were used to determine the quality of life, physical activity and sedentary behaviour respectively. Participants were assessed at pre and post-intervention.

The SF-36v2 tool contains 36 questions and eight subscales measuring elements such as physical functioning, role limitations due to physical health, and pain, and contains general health subscales consisting of the total physical score, the mean score of emotional well-being, social functioning, role limitations due to emotional problems, energy/fatigue, and total mental score. The sub-dimensions scores ranged 0–100 points with higher scores indicating a better quality of life [15]. The SF-36 is a valid and reliable tool [16, 17]. The short version of IPAQ is well recognised [18, 19] tool to assess PA levels [15, 19] that has been previous validated [20]. WSQ is a validated tool used to determine time spent sitting during average workday and non-workdays [21].

A questionnaire administered 1-month post-intervention asked participants about their reasons for participating/not participating in the study or for not completing the programme; what they gained from the programme; and whether they would continue the intervention, even after the study period.

### Statistical analysis

All data were analysed and coded using SPSS software version 21.0 (SPSS, Inc. Chicago, IL, USA). Continuous variables are presented as the mean and standard deviation, and categorical variables as the number and percentages. The Health Survey SF36v2 was scored using Health Outcomes Scoring Software 5.1 (QualityMetric, Inc., Lincoln, RI, USA). The IPAQ scores are presented as total minutes spent in the metabolic equivalent (MET) of task.

Normality assumptions were tested before statistical analysis [22, 23]. All continuous variables were tested for normality using the Shapiro–Wilk test, and log transformation was applied to IPAQ total activity MET-minutes per week as it was not normally distributed. An independent sample *t*-test was used to determine differences in PA, quality of life, and sedentary behaviour between categorical variables (e.g. sex and marital status). One-way analysis of variance was used to compare the same scores for categorical variables with more than two categories (e.g. ethnicity). Post hoc comparisons were conducted using Bonferroni correction.

To determine the intervention’s effectiveness amongst 54 participants who provided complete (i.e. pre-intervention, intervention, and post-intervention) pedometer data, a linear mixed model was used that incorporated step count as the dependent variable. Bonferroni correction was applied for all pairwise comparisons in this case.

### Ethical considerations

This study was approved by Qatar Anti-Doping Lab Ethics Committee (Doha, Qatar; approval no: E2017000215). Participation was voluntary and their personal information remained confidential. Respect for culture was of utmost importance.

## Results

### Demographics

The participants’ sociodemographic features pre-intervention and post-intervention are presented in Table 1. At pre-intervention, the percentage of men was greater than that of women (70 and 30%, respectively). Participants at pre-intervention were mostly 35–44 years old (45%), married (84.0%) white (43%) and most holding a bachelor’s degree or higher (78%). There was little change in the sociodemographic data from pre to post intervention. The demographic information of subjects who provided the valid pedometer data is also mentioned in Table 1.

### Questionnaire data on physical activity

Based on the IPAQ, the total PA was higher post 3-month intervention (183.6 ± 110.9 min/week) compared to pre-intervention (161.7 ± 108.7 min/week). The SF36v2 was not any different in the post-intervention sample compared to the pre-intervention sample (Table 2).

**Table 2:** Summary of mean scores of SF36 v2, IPAQ, and WSQ among participants from the pre-test sample and post-test sample

Variable	Cross-sectional sample Pre (*n* = 212)	Cross-sectional sample Post (*n* = 194)
SF36 v2 (Health Survey)	Mean ± SD	Mean ± SD
Physical Component Summary	53.6 ± 6.6	54.5 ± 6.3
Mental Component Summary	49.1 ± 9.0	49.9 ± 9.3
Physical functioning	87.5 ± 18.6	88.6 ± 19.2
Role Physical	81.0 ± 24.1	85.6 ± 20.8
Bodily pain	77.7 ± 20.1	78.4 ± 19.9
General health	73.2 ± 18.3	76.4 ± 17.0
Vitality	66.9 ± 17.3	68.3 ± 16.5
Social functioning	77.2 ± 23.4	79.8 ± 22.0
Role Emotional	82.0 ± 21.4	84.2 ± 22.5
Mental health	73.7 ± 17.7	74.9 ± 17.5
International Physical Activity Questionnaire (IPAQ)	Mean ± SD	Mean ± SD
Total activity (min/week)	161.7 ± 108.7	183.6 ± 110.9
Vigorous (MET-minutes per week)	1221.7 ± 1458.2	1439.2 ± 1630.7
Moderate (MET-minutes per week)	718.4 ± 1088.9	804.4 ± 978.3
Walking (MET-minutes per week)	1414.6 ± 1368.6	1466.4 ± 1246.3
Total (MET-minutes per week)	3354.7 ± 2929.9	3710.0 ± 2873.1
IPAQ Physical activity level n(%)	n (%)	n (%)
Low	46 (21.7)	22 (11.3)
Moderate	50 (23.6)	55 (28.4)
High	116 (54.7)	117 (60.3)
Workforce sitting questionnaire (WSQ) Time spent sitting (min/day)	Mean ± SD	Mean ± SD
On Workday		
For transport	96.7 ± 83.6	90.5 ± 101.1
At work	208.3 ± 140.6	252.7 ± 153.6
Watching TV	65.0 ± 69.3	66.1 ± 65.1
Using computer at Home	101.4 ± 94.5	95.1 ± 105.7
Other leisure activities	48.1 ± 61.4	39.9 ± 62.3
On a Non workday		
For transport	74.2 ± 78.7	75.9 ± 73.5
At work	90.3 ± 108.5	93.9 ± 123.3
Watching TV	126.4 ± 111.6	132.2 ± 110.9
Using computer at Home	123.1 ± 100.6	115.9 ± 102.2
Other leisure activities	153.3 ± 113.2	142.9 ± 108.2

The total MET-minutes per week post-intervention was higher 3710.0 ± 2873.1 when compared to 3354.7 ± 2929.9 min per week pre-intervention. More participants engaged in more moderate and high intensity physical activity following the intervention than they did pre. Although time spent sitting was similar while watching TV, post intervention participants spent on average 44 min more per day sat down at work.

### Physical activity levels

Fifty-four participants provided complete pedometer data for the three time points of pre-intervention, during intervention, and post-intervention (Table 3). The average steps were significantly increased during intervention compared to pre-intervention (*p* = 0.048). The difference at post-intervention was statistically similar to pre-intervention. Throughout the study, male staff were generally more active than female staff (average, 10,440 steps vs. 6694 steps) (*p* = 0.024). While there was an overall increase in step count during the intervention, these changes were not associated with age, sex or BMI status.

**Table 3:** Summary of average steps taken (±SE) before, during, and after the intervention in relation to age, sex, and BMI status (*n* = 54)

	Time Points of the 3-month walking intervention					
	Pre-intervention		During		Post-intervention	
	Steps (Mean)	Aerobic Steps (Mean)	Steps (Mean)	Aerobic Steps (Mean)	Steps (Mean)	Aerobic Steps (Mean)
Overall	7890 ± 713	1359 ± 399	9270 ± 672*	2275 ± 358	8998 ± 683	2109 ± 369
Sex						
Female (*n* = 23)	5104 ± 1054	261 ± 603	6694 ± 990	953 ± 543	6886 ± 1037	1193 ± 590
Male (*n* = 30)	9357 ± 911	1937 ± 521	10,405 ± 853	2857 ± 463	9900 ± 855	2501 ± 464
Age						
<=40 (*n* = 21)	7447 ± 1205	1149 ± 697	10,440 ± 1093	3052 ± 588	8722 ± 1101	1756 ± 596
> 40 (0n = 32)	8026 ± 902	1423 ± 493	8767 ± 863	1940 ± 454	9103 ± 880	2245 ± 472
BMI						
Normal (*n* = 21)	6058 ± 1127	532 ± 626	8722 ± 1070	2376 ± 571	8432 ± 1110	1619 ± 612
Overweight (*n* = 32)	8950 ± 926	1838 ± 523	9616 ± 872	2211 ± 463	9270 ± 872	2345 ± 467

*p = 0.048 when compared to baseline

## Discussion

The levels of physical inactivity in the Eastern Mediterranean region are the highest in the world and while certain factors pose a challenge to physical activity engagement more needs to be done to try and engage the population and limit the increasing burden of non-communicable disease. Workplace wellness and health interventions have been posed as a viable intervention, and the main findings from our study highlights that a 3 month controlled pedometer based walking programme is a feasible and effective tool in achieving this. The adult hospital workers participating in the study increased their daily number of steps by 1380 with this value still larger post intervention than compared to pre. Female workers were more inactive than male workers at pre-intervention, which concurs with the worldwide trend reported by the WHO [4] The pre-intervention BMI data indicated most hospital staff had a higher risk of various metabolic and disease outcomes, although the participants classed as overweight according to BMI completed on average 894 steps more per day than their non-overweight colleagues.

### Effectiveness of workplace walking programme

At pre-intervention in averaging 5104 steps per day women were classified as being ‘low active’ while the men ‘moderately active’ (9357 steps). Twenty-six percent of study participants with complete pedometer data had a significant increase in the overall step count during the 3-month workplace walking intervention.

The average step count of both sexes increased significantly from pre-intervention to during the intervention and post-intervention. Men were in the ‘active’ and ‘moderate’ categories during intervention and post-intervention, respectively. Women were in the ‘low active’ category pre-intervention and post-intervention; however, their average step count increased by 34%, which is similar to the 23% increase reported by Bravata et al. [24]. In a pedometer based study of Qatari females Sayegh et al. (2016) [7] suggest that certain cultural restrictions and other social factors may be the reason for low adoption of physical activity, with the authors suggesting exercise not widely accepted among this population.

### Prevalence of occupational sitting

In our sample, according to the WSQ, on average men spend 9.3 ± 4 h and women 8.3 ± 3.9 h sitting over working day. While a 2010 systematic review including 43 articles suggested there was limited evidence of a relationship between occupational sitting and health risks the wide variety of designs, methodology and outcome data makes it difficult to confidently draw conclusions [25]. The findings from the present study should still be a cause for concern given research looking into general sedentary behaviour, where sitting times between 7 and 12 h per day have been linked to an increased risk in all-cause mortality [26–29]. For example Chau et al. [26] report a 65% higher risk of all-cause mortality amongst adults with > 10 h/day of total sitting time, compared with individuals with < 4 h/day of total sitting time. While asserting the longitudinal health benefits of workplace walking programmes was out of the scope of the current research, continued research needs to be conducted to fully elucidate the impact of workplace sitting on overall health.

### Racial differences

Given the global make up of our organisation we were able to investigate the disparity in physical activity between employees of different races. In our study, compared with Arab and Asian participants, the white participants had better physical functioning, general health, emotional scores, and social functioning. Wu and Schimmele [30] report that the health status is often a function of behaviour and personal attitudes. Evidence also demonstrates that racial/ethnic health disparities parallel differences in health behaviours [31, 32].

The mean physical component summary (PCS) and mental component summary (MCS) scores pre-intervention (53.6 and 49.1, respectively) and post-intervention (54.5 and 49.9, respectively) were comparable with those reported by Mansi et al. [33] (e.g. pre-intervention PCS and MCS scores, 49.3 and 50.3, respectively). Bjorner et al. [34] demonstrated that a one-point decrease in the scores of any of the eight components of the SF-36v2 tool corresponded to a 1.02–1.04 relative risk of hospitalisation and a 1.07–1.12 relative risk of being unable to work. Unfortunately, the pre- and post-intervention health survey scores were not compared because it was impossible to determine whether the scores were from the same participant.

### Barriers to participation

Seventy-eight percent of participants did not participate in the SIH programme. Some major reasons cited were lack of time, family obligations, and lack of motivation. Other reasons were lack of energy, travel, social influence, and weather conditions. Hospital employees in a study by Blake and Batt [35] also reported these factors as barriers. Hot weather as a barrier to achieving walking goals in this study was consistent with research indicating seasonal variations in pedometer use and daily accumulated step count [36]. The 20% of participants who finished the programme reported feeling good; having less stress; gaining healthier habits; improved physical fitness; increased well-being, self-image, and self-esteem; increased work productivity; weight loss; and decreased illness and injury. Seventeen percent of participants indicated they would continue the programme.

### Limitations

The response rate during pre-intervention and post-intervention surveys was very low as was the number of participants who were willing to share their pedometer online. This study implemented a repeated measures design whereby outcome measures were only assessed at pre-intervention, 3-months and post intervention. Whether the pre post-intervention questionnaire data were completed by the same person who uploaded the data, or whether all people participated in the intervention was challenging to determine. Therefore, unfortunately comparing these data was invalid and not feasible.

## Conclusion

Although during the intervention period there were promising results of higher physical activity among hospital workers, this decreased post intervention. Moreover, this did not reach the WHO recommendation of 10,000 steps per day for working adults [5]. Participants expressed positive responses to the intervention but an even smaller percentage expressed they would continue the intervention after the study. Therefore, encouraging participation and maintaining motivation in the long term amongst workers in a work-based PA programme is challenging.

## Data Availability

The datasets used and/or analysed during the current study are available from the corresponding author on request.

## Abbreviations

BMI: Body Mass Index
EMR: East Mediterranean Region
IPAQ: International Physical Activity Questionnaire
MCS: Mental Component Summary
MET: Metabolic Equivalent
PA: Physical Activity
PCS: Physical Component Summary
SF-36v2: The Health Survey Short Form-36, Version 2
SIH: Step Into Health
WHO: World Health Organization
WSQ: Workforce Sitting Questionnaire
